# Evaluation of The Relationship among The Levels of SIRT1 and SIRT3 with Oxidative Stress and DNA Fragmentation in Asthenoteratozoospermic Men

**DOI:** 10.22074/IJFS.2020.134692

**Published:** 2021-03-11

**Authors:** Abolfazl Nasiri, Asad Vaisi-Raygani, Zohreh Rahimi, Mitra Bakhtiari, Fariborz Bahrehmand, Amir Kiani, Hadi Mozafari, Tayebeh Pourmotabbed

**Affiliations:** 1 Students Research Committee, Kermanshah University of Medical Sciences, Kermanshah, Iran; 2 Fertility and Infertility Research Center, Health Technology Institute, Kermanshah University of Medical Sciences, Kermanshah, Iran; 3 Medical Biology Research Center, Health Technology Institute, Kermanshah University of Medical Sciences, Kermanshah, Iran; 4 Regenerative Medicine Research Center, Kermanshah University of Medical Sciences, Kermanshah, Iran; 5 Department of Microbiology, Immunology, and Biochemistry, University of Tennessee Health Science Center, Memphis, TN, USA

**Keywords:** DNA Fragmentation, Infertility, Oxidative Stress, SIRT1, SIRT3

## Abstract

**Background:**

Reactive oxygen species (ROS) play a crucial role in etiology of DNA fragmentation and lipid peroxi- dation in sperm, leading to infertility in men. The silent information regulators SIRT1 and SIRT3 are members of the sirtuins protein family known to be involved in cancer genetics, aging and oxidative stress responses. The aim ofthis study is to determine the correlation between SIRT1 and SIRT3 with antioxidants, oxidative stress biomarkers, and DNA fragmentation in the semen of asthenoteratozoospermic and normozoospermic men.

**Materials and Methods:**

In this case-control study, after spermogram analysis the specimens were divided into two groups, normozospermic (n=40) and asthenoteratozoospermic (n=40), according to World Health Organization (WHO) standards. Sperm DNA fragmentation was evaluatedusing the sperm chromatin dispersion (SCD) test.Catalase activity was measured using the Aebi spectrophotometeric method. Total antioxidant capacity (TAC) level and super- oxide dismutase (SOD) activitywere measured by using commercially available colorimetric assays. Enzyme-linked immune sorbent assay (ELISA) was used to measure SIRT1 and SIRT3 protein levels of seminal plasma. Malondial- dehyde (MDA) level in seminal plasma was determined by high-performance liquid chromatography (HPLC).

**Results:**

The asthenoteratozoospermic group had significantly lower catalase and SOD activities and TAC levels in comparison with the normozoospermic group (P<0.001).The percentage of DNA fragmentation and MDA level in the asthenoteratozoospermic group were remarkably higher than in the normozoospermic group. The SIRT1 and SIRT3 protein levels in seminal plasmawere remarkably lower in asthenoteratozoospermic group than the normozoospermic group (P<0.001).

**Conclusion:**

The results of this study suggest that SIRT1 and SIRT3 protein levels are negatively correlated with oxidative stress and DNA fragmentation in semen. The low levels of SIRT1 and SIRT3 in asthenoteratozoospermic men may lead to an increase in oxidative stress, DNA fragmentation, and lipid peroxidation that eventually result in immotile and immature spermatozoa (asthenoteratozoospermia).

## Introduction

Up to 15 percent of couples are infertile and male infertility includes more than 40% of all infertility cases (1). DNA fragmentation and membrane lipid peroxidation have been regarded as influential factors inthe etiology of male infertility. Many studies have attributed oxidative stress to the DNA fragmentation in men. Many studies suggest that ROSa clue in etiology of DNA fragmentation and lipid peroxidation. In pathologic conditions, the generation of ROS exceeds the enzymatic [glutathione peroxidase (GPx), superoxide dismutase (SOD) and catalase] and non-enzymatic (glutathione and vitamins A, E, C) antioxidant capacities in seminal plasma, resulting in lipid peroxidation and DNA fragmentation (2, 3).

Sirtuins family, which are also referred to as the silent information regulators, are nicotinamide adenine dinucleotide (NAD)-dependent class III histone deacetylase enzymes, which are present in a wide range of microorganisms from bacteria to humans (4). Generally, sirtuins are involved in various biological and physiological functions, including resistance to oxidative stress, DNA repair, apoptosis and the control of metabolic enzymes (5-7). In mammals, seven sirtuins have been discovered, ranging from SIRT1 to SIRT7. SIRT1 and SIRT2 exist in the cytoplasm and nucleus, while SIRT6 and SIRT7 are located only in the nucleus. SIRT3, SIRT4, and SIRT5 are known as mitochondrial sirtuins (8).

Among mammalian sirtuins, the SIRT1 and SIRT3 are well characterizedand are reported to play a role in oxidative stress responses (6, 9, 10). SIRT1 that regulates proinflammatory mediators by deacetylating histone and non-histone proteins,enhances the levels of SOD2, GPx and catalase through activation of p53, FOXO3a and PGC1 transcription factors (11, 12). SIRT3 is the major mitochondrial deacetylase that regulates the enzymatic activity of critical metabolic enzymes in biochemical pathways, such as the tricarboxylic acid (TCA) cycle and electron transport chain, and it reduces oxidative stress. 

Studies have shown that deacetylation of SOD2 and catalase by SIRT3 increases their catalytic activities. In addition, the catalytic activity of SOD2 is diminished when SIRT3 is deleted (13). Thus, SIRT3 regulates the levels of cytoplasmic and mitochondrial ROS and regulates the complex III of chain reaction, one of the main source of ROS production (14).

Testes express high levels of sirtuins (15). It has been shown that SIRT1 is involved in male germ cell development during the spermatogenesis (16, 17) and that Sirt1 knockout mice become completely infertile. A decrease in SIRT1 expression causes defects in acrosome formation and consequently changes in sperm morphology (18). However, the relationship between SIRT1 and SIRT3, especially, with oxidative stressin the semen and male infertility is largely unclear.Considering the antioxidant roles of SIRT1 and SIRT3, we hypothesized that SIRT1 and SIRT3 may play a contributory role in the ROS damage (lipid peroxidation, and DNA fragmentation) of sperms, and the aim of this study was to determine the association of SIRT1 and SIRT3 expression with oxidative stress, lipid peroxidation, and DNA fragmentation in asthenoteratozoospermia compared to normozoospermia.

## Materials and Methods

### Study participants and sample collection

The present research is a case-control study. The study was confirmed by the Research Ethical Committee of Kermanshah University of Medical Sciences (IR. KUMS.REC.1397.353). Informed consent was received from all participants before sample collection. The exclusion criteriawere leukocyte concentration >0.8×10^6^ per milliliter of ejaculation (highly increased leukocyte count leads to the formation of oligozo and azospermia in the samples. Specimens with cytoplasmic droplets >50% and round sperm>10^6^ per milliliter, samples with an infection such as bacteria or viruses, hyperviscous samples, samples from subjects with a history of smoking and/or diseases (varicocele, diabetes mellitus, hypertension, and chronic diseases) were also excluded from the study. Semen samples from 80 healthy donors, aged between 22-45 years, were obtained from the individuals referred to Moatazedi Infertility Center by masturbation after 3 days of sexual abstinence. The semen samples were analyzed macroscopically (physical appearance, volume, and pH) and microscopically (count, motility, and morphology) according to WHO (2010) recommendations within 1 hour after sample collection (19). In addition to spermogram, morphology was determined by Diff-Quik staining and was graded according to the Kruger’s strict criteria and cutoff value established by WHO (2010) guideline. Motility was determined and categorized (progressive spermatozoa, non-progressive spermatozoa and immotile spermatozoa) by movements of 100spermatozoa. After spermogram analysis (especially morphology and motility). Specimens were divided into two groups, normozospermic (n=40) and asthenoteratozoospermic (n=40) based on the WHO standards.

One hundred microliters of each semen sample was used for DNA isolation and the seminal plasma was separated from the remaining portion of the semen samplesby centrifugation at 600 g for 10 minutesand stored at -80°C for subsequent measurements ofoxidative stress biomarkers [malondialdehyde (MDA), catalase, SOD, and total antioxidant capacity (TAC)] and SIRT1 and SIRT3 pr

### Antioxidants and oxidative stress biomarkers

### Measurement of malondialdehyde

The level of lipid peroxidation in seminal plasma was determined according to the MDA levels, which were determined by reverse phase high-performance liquid chromatography (HPLC) (Agilent Technologies1200 Series) with a fluorescence detector using EC 250/4.6, Nucleodur100–5 C18ec column (Macherey-Nagel, Duren, Germany); mobile phase 60:40 V/V of methanol: buffer (50 mM potassium monobasicphosphate buffer with pH=6.8); detection, Ex515-Em555 (20).

### Measurement of catalase activity

The activity of seminalplasma catalase was determined according to Aebi et al. (21). Catalase degrades hydrogen peroxide (H_2_O_2_). The basis of this method is a direct measurement of the reduction of H_2_O_2_ absorption by catalase activity at 240 nm wavelength, which is ultimately expressed as Enz. Ut/l.

### Measurement of superoxide dismutase activity

The activity of seminalplasma SOD was determined by colorimetric assay (Kiazist SOD kit, Kiazist, Iran). The main principle in the management of this laboratory method is the production of superoxide radicals by xanthine oxidase. These radicals react with resazurin to produce resorufindye, which is detectable at 570 nm (Ex/Em=575/585). The SOD activity is then calculated as the percentage of reduction in resazurin production. A unit of SOD activity was described as the amount of SOD reducing production of resazurin by 50%. 

### Measurement of total antioxidant capacity

TAC was measured by Kiazist TAC kit (Kiazist, Iran). This protocol employs the ferric reducing antioxidant power (FRAP) method, which is relying on the reduction of the ferric tripyridyltriazine to the ferrous tripyridyltriazine along with a color production visible at 450 nm.

### Measurement of SIRT1 and SIRT3

Concentrations of SIRT1 and SIRT3 in the semen samples were measured by enzyme-linked immune sorbent assay (ELISA) kit (Eastbiopharm, Hangzhou, USA). 

### Assessment of sperm DNA fragmentation

Halosperm kit INDAS Laboratories was used to evaluated DNA fragmentation using the sperm chromatin dispersion (SCD) test. In an SCD test, the spermatozoa containing non-fragmented DNAdisplaya large and spotty halo dispersed DNA loops, while the spermatozoa with fragmented DNA show very small or no halos (22).

### Statistical analysis

The data are shown as mean ± standard deviation (SD) and analyzed by using statistical software SPSS (version 16, Chicago, IL). The normality of variables are analyzed by Kolmogorov-Smirnov test. The student’s t test was used for comparisons of the parameters in asthenotratozoospermic and normozoospermic groups. Pearson’s correlation coefficient was evaluated for correlation among the variables. A probability (P) value less than 0.05 was considered statistically significant.

## Results

The results of the variable parameters including age, body mass index (BMI) and routine semen analysis are summarized in Table1. As expected, sperm morphology in asthenoteratozoospermic group was abnormal and sperm motility in asthenoteratozoospermic group was remarkably lower than in the normozoospermic group (P<0.001). 

### Level of antioxidants and oxidative stress biomarkers in seminal plasma

Asthenoteratozoospermic groups had lower levels of catalase (P<0.001), SOD (P<0.001), and TAC (P<0.001) and higher levels of MDA (P<0.001) in seminal plasma compared to the normozoospermic group (Table 2

**Table 1:** Baseline characteristics of the age, BMI and semen parameters


Parameters	Groups		P value
	Normozoospermia n=40	Asthenoteratozoospermia n=40	

Age (Y)	34.67 ± 5.37	35.65 ± 4.82	0.396
BMI (Kg M^-^^2^)	27.40 ± 3.20	26.42 ± 3.55	0.201
pH	7.54 ± 0.11	7.50 ± 0.14	0.176
Volume (ml)	3.35 ± 0.21	3.31 ± 0.52	0.821
Concentration 10^6^ per ml)	43.37 ± 9.41	35.72 ± 5.62	0.001^*^
Motility (%)	23.37 ± 2.54	16.75 ± 2.65	0.001^*^
Morphology (%)	7.00 ± 1.12	3.9 ± 1.2	0.001^*^


The data are shown as mean ± SD. * ; P value<0.05, significant difference among the groups and BMI; Body mass index.

**Table 2:** Comparison of seminal plasma antioxidants and oxidative stress biomarkers


Parameters	Groups		P value
	Normozoospermian=40	Asthenoteratozoospermia n=40	

MDA (µmol/L)	0.75 (0.52-1.12)	2.12 (1.51-3.01)	0.001^*^
Catalase (U/ml)	20.18 (13.36-30.06)	9.04 (2.22-13.36)	0.001^*^
SOD (U/ml)	23.48 (20.23-26.19)	12.08 (9.52-14.28)	0.001^*^
TAC (mM)	2.14 (1.84-2.40)	1.20 (0.97-1.40)	0.001^*^


Data are presented as median and interquartile range (IQR). *; P<0.05, significant difference among the groups, MDA; Malondialdehyde, SOD; Superoxide dismutase, and TAC; Total antioxidant capacity

### DNA fragmentation

**Fig.1:**
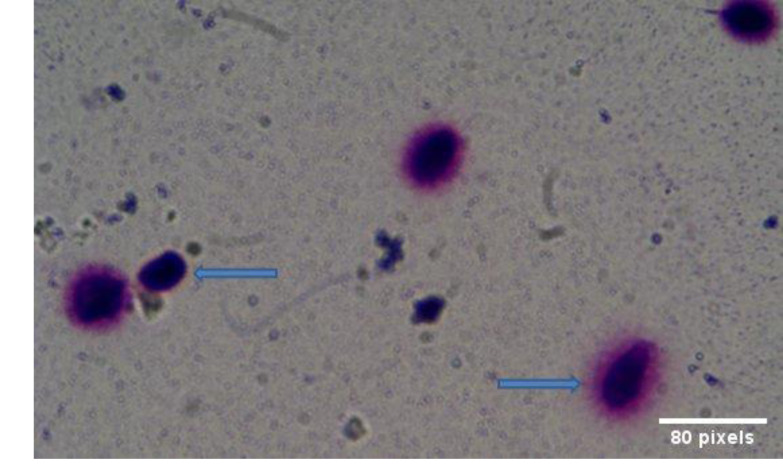
Visualization of DNA fragmentation in sperm samples using the SCD test. Photographs were taken under light microscopy (×50) (Olympus BX- 40, Olympus U-RFL-T, and Tokyo, Japan). The spermatozoa with DNA fragmentation show the lack or small haloes of chromatin (left), whereas these haloes were detected as larger spheres in spermatozoa with no DNA fragmentation (right). SCD; Sperm chromatin dispersion.

**Fig.2:**
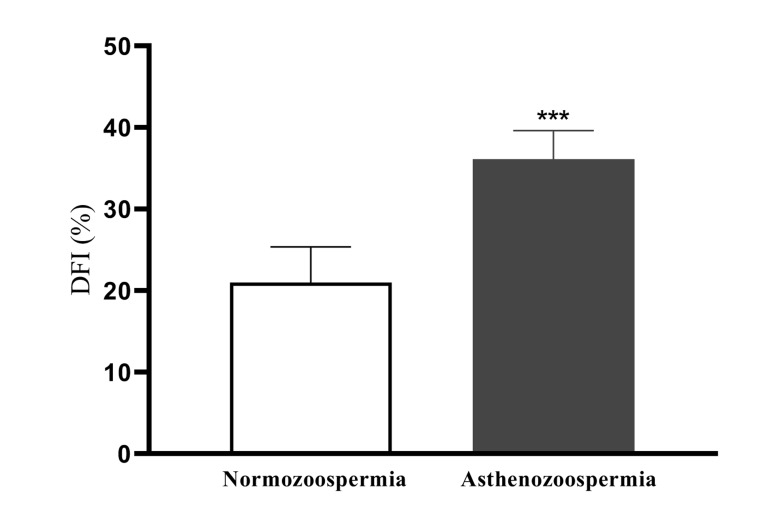
DNA fragmentation in normosospermic (n=40) and asthenoteratozoospermic (n=40) sperm samples using the sperm chromatin dispersion (SCD) test. ***; P<0.001.

The spermatozoa with DNA fragmentation had small haloes or no haloes at all, whereas spermatozoa with no DNA fragmentation showed large haloes of chromatin, as shown in Figure 1. The percentages of spermatozoa cells with fragmented DNA were 20.97% (17.00-24.00) and 36.1% (34-38.75) in normozoospermicand and asthenotratozoospermic groups, respectively. DNA fragmentation was significantly (P<0.001) higher in the asthenoteratozoospermic group compared to the normozoospermic group (Fig.2).

### SIRT1 and SIRT3

The protein levels ofSIRT1 and SIRT3 weresignificantly (P<0.001) low in the asthenoteratozoospermic group compared to the normozoospermic group. The amount of SIRT1 protein in the asthenoteratozoospermic and normozoospermic groups were 5.1 ± 0.59 ng/ml and 7.02 ± 0.62 ng/ml , respectively. The amounts of SIRT3 protein in asthenoteratozoospermic and normozoospermic groups were 3.20 ± 0.21 ng/ml and 4.28 ± 0.32 ng/ml, respectively (Figes.3, 4).

**Fig.3:**
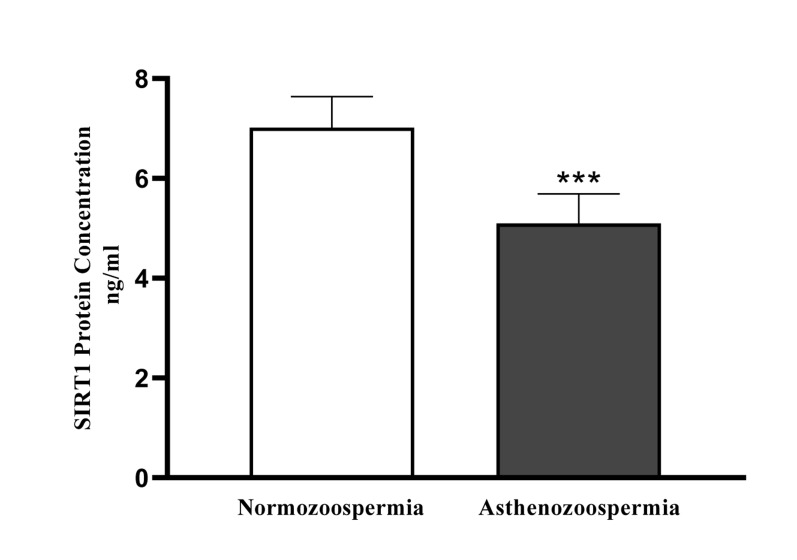
Sperm SIRT1 level (ng/ml) of normozoospermic (n=40) and asthenoteratozoospermic (n=40) men were measured by enzyme-linked immune sorbent assay (ELISA) test. ***; P<0.001.

**Fig.4:**
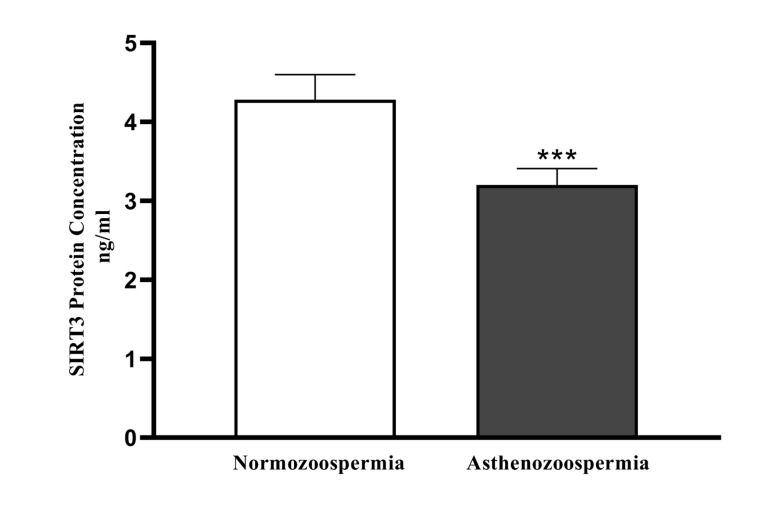
SpermSIRT3 level (ng/ml) of normozoospermic (n=40) and asthenoteratozoospermic (n=40) men were measured by enzyme-linked im- mune sorbent assay (ELISA) test. ***; P<0.00

## Discussion

This study demonstrated that: i. There is a significant elevation inMDA, oxidative stress biomarkers, and the percentage of DNA fragmentation, as well as a decrease in antioxidant biomarkers, such as catalase, SOD activity, and TAC levels in asthenoteratozoospermic specimens compared with normozoospermic specimens; ii. SIRT1 and SIRT3 proteins exist in the seminal plasma; and iii. Both SIRT1 and SIRT3 levels in seminal plasma are negatively correlated with oxidative stress and DNA fragmentation.

Although association of seminal levels of SIRT1 and SIRT3 with male infertility at this point is unclear, the results of this study suggest that low SIRT1 and SIRT3 protein levelsin seminal plasma correlate with a high concentration of ROS andabnormal morphology and motility of sperms in asthenoteratozoospermia. Seminal plasma is involved in providing nutrition for the maintenance of spermatozoa. Increased ROS production in the seminal plasma leads to reduced sperm metabolism and motility, resulting in infertility (23, 24). Studies have shown that under mild oxidative stress, the expression and activation of SIRT1 is increased, while high and harsh ROS results in increased destruction and inactivation of SIRT1 (25). These results in combination with our findings, suggest that the relationship between SIRT1 and SIRT3 with oxidative stress is reciprocal. These findings suggest that high oxidative stress leads to a decrease in the amount and activity of SIRT1 and SIRT3 through i. Inducing the expression of sirtuins, ii. Altering protein interactions with sirtuins post-translationally, and iii. Changing NAD levels. Ultimately, the decrease in SIRT1 and SIRT3 leads to an increase in ROS levels (26). In addition, SIRT1 and SIRT3 are involved in ROS production due to their effects on the expression and activation of antioxidants such as SOD, catalase and GPx (11-14, 26).

Lipid peroxidation plays a major role in the quality of sperm. In this study we have shown that there is a direct relationship between increased levels of MDA and abnormal of sperm morphology and motility. This is similar to several studies showing that lipid peroxidation changes the morphology of the sperm and reduces its concentration and motility (3, 27). However, Suleiman et al. (28) showed no significant correlation between the increased levels of MDA and concentration and motility of sperms in seminal plasma.

The present study has also demonstrated that there is a direct relationship between increased activity of SOD with sperm morphology and motility improve, suggesting that there is a direct relationship between the activity of SOD and the optimal quality of sperms. This is consistent with the studies of Macanovic et al. (29) and Khosrowbeygi et al. (30), showing that the activity of catalase and the level of TAC are low in the seminal plasma of infertile men and these phenomena are directly related to poor motility and morphology of sperms. However, in several studies, no significant relationship between the SOD or catalase activity and the quality of sperms was found (31, 32). 

Aktan et al. (33) and Atig et al. (34) stated that elevation of oxidative stress and a decline in antioxidant defense in seminal fluid are the most important factors in DNA fragmentation. DNA fragmentation reduces sperm quality and therefore, it increases the incidence of infertility in men (7, 35). The results of the present study show that there is a direct relationship between DNA fragmentation, increase in MDA, and the reduction of antioxidants (catalase, SOD, and TAC). In addition, we have found that catalase, SOD, and TAC levels are positively correlated with low SIRT1 and SIRT3 levels in asthenoteratozoospermia. In addition, SIRT1 is involved in male germ cell development during the spermatogenesis (16, 17) and thus in *Sirt1* knockout mice, they became completely infertile. Lower level of SIRT1 causes defects in acrosome formation and consequently changes in sperm morphology (18). These data suggest that reduction in SIRT1 and SIRT3 levels in seminal plasma lead to antioxidants reduction, resulting in an increase in ROS and DNA fragmentation and thus, affecting semen quality and reducing sperm motility and changing sperm morphology in asthenoteratozoospermia.

## Conclusion

This study demonstrated that SIRT1 and SIRT3 protein levels and the antioxidant biomarkers are reduced, while the amount of oxidative biomarkers and DNA fragmentation are increased in asthenoteratozoospermia. These changes lead to defects in the morphology and motility of sperms in the semen and thus, infertility in men. Although in idiopathic infertility and assessing the health of reproductive systems this finding is important, further molecular and biochemical studies are needed to determine the exact mechanisms, through which SIRT1 and SIRT3 affect fertility. Also, investigating the correlation between the results of the present study and the success rate of assisted reproductive methods or *in vitro* fertilization (IVF) may be helpful in predicting the outcomes of such methods.

## References

[1] Agarwal A, Virk G, Ong C, du Plessis SS (2014). Effect of oxidative stress on male reproduction. World J Mens Health.

[2] Homa ST, Vassiliou AM, Stone J, Killeen AP, Dawkins A, Xie J (2019). A comparison between two assays for measuring seminal oxidative stress and their relationship with sperm DNA fragmentation and semen parameters. Genes (Basel).

[3] Atig F, Raffa M, Ali HB, Abdelhamid K, Saad A, Ajina M (2012). Altered antioxidant status and increased lipid per-oxidation in seminal plasma of tunisian infertile men. Int J Biol Sci.

[4] Sauve AA, Youn DY (2012). Sirtuins: NAD(+)-dependent deacetylase mechanism and regulation. Curr Opin Chem Biol.

[5] Chang H-C, Guarente L (2014). SIRT1 and other sirtuins in metabolism. Trends Endocrinol Metab.

[6] Singh CK, Chhabra G, Ndiaye MA, Garcia-Peterson LM, Mack NJ, Ahmad N (2018). The role of sirtuins in antioxidant and redox signaling. Antioxid Redox Signaling.

[7] Tatone C, Di Emidio G, Barbonetti A, Carta G, Luciano AM, Falone S (2018). Sirtuins in gamete biology and reproductive physiology: emerging roles and therapeutic potential in female and male infertility. Hum Reprod Update.

[8] Nasiri A, Sadeghi M, Vaisi-Raygani A, Kiani S, Aghelan Z, Khodarahmi R (2020). Emerging regulatory roles of mitochondrial sirtuins on pyruvate dehydrogenase complex and the related metabolic diseases. Biomed Res Ther.

[9] Carnevale I, Pellegrini L, D'Aquila P, Saladini S, Lococo E, Polletta L (2017). SIRT1-SIRT3 axis regulates cellular response to oxidative stress and etoposide. J Cell Physiol.

[10] Ansari A, Rahman MS, Saha SK, Saikot FK, Deep A, Kim KH (2017). Function of the SIRT3 mitochondrial deacetylase in cellular physiology, cancer, and neurodegenerative disease. Aging Cell.

[11] Waldman M, Cohen K, Yadin D, Nudelman V, Gorfil D, LaniadoSchwartzman M (2018). Regulation of diabetic cardiomyopathy by caloric restriction is mediated by intracellular signaling pathways involving ‘SIRT1 and PGC-1α’.Cardiovasc. Diabetol.

[12] Hori YS, Kuno A, Hosoda R, Horio Y (2013). Regulation of FOXOs and p53 by SIRT1 modulators under oxidative stress. PLoS One.

[13] Tao R, Coleman MC, Pennington JD, Ozden O, Park SH, Jiang H (2010). Sirt3-mediated deacetylation of evolutionarily conserved lysine 122 regulates MnSOD activity in response to stress. Mol Cell.

[14] Bell EL, Guarente L (2011). The SirT3 divining rod points to oxidative stress. Mol Cell.

[15] Michishita E, Park JY, Burneskis JM, Barrett JC, Horikawa I (2005). Evolutionarily conserved and nonconserved cellular localizations and functions of human SIRT proteins. Mol Biol Cell.

[16] Coussens M, Maresh JG, Yanagimachi R, Maeda G, Allsopp R (2008). Sirt1 deficiency attenuates spermatogenesis and germ cell function. PLoS One.

[17] Niu B, Wu J, Mu H, Li B, Wu C, He X (2016). miR-204 regulates the proliferation of dairy goat spermatogonial stem cells via targeting to Sirt1. Rejuvenation Res.

[18] Liu C, Song Z, Wang L, Yu H, Liu W, Shang Y (2017). Sirt1 regulates acrosome biogenesis by modulating autophagic flux during spermiogenesis in mice. Development.

[19] Sanità Omd (2010). WHO laboratory manual for the examination and processing of human semen.5th ed.Geneva.Switzerland. World Health Organization.

[20] Najafi K, Ahmadi S, Rahpeyma M, Khazaie H, Vaisi-Raygani A, Moini A (2017). Study of serum malondialdehyde level in opioid and methamphetamine dependent patients. Acta Med Iran.

[21] Aebi H, Wyss SR, Scherz B, Skvaril F (1974). Heterogeneity of erythrocyte catalase II.Isolation and characterization of normal and variant erythrocyte catalase and their subunits. Eur J Biochem.

[22] The Practice Committee of the American Society for Reproductive Medicine (2013). The clinical utility of sperm DNA integrity testing: a guideline. Fertil Steril.

[23] Bisht S, Faiq M, Tolahunase M, Dada R (2017). Oxidative stress and male infertility. Nat Rev Urol.

[24] Moradi M-n, Karimi J, Khodadadi I, Amiri I, Karami M, Saidijam M, et al (2018). Evaluation of the p53 and Thioredoxin reductase in sperm from asthenozoospermic males in comparison to normozoospermic males. Free Radical Biol Med.

[25] Yang Y, Fu W, Chen J, Olashaw N, Zhang X, Nicosia SV (2007). SIRT1 sumoylation regulates its deacetylase activity and cellular response to genotoxic stress. Nat Cell Biol.

[26] Santos L, Escande C, Denicola A (2016). Potential modulation of sirtuins by oxidative stress.Oxid Med Cell Longev.

[27] Benedetti S, Tagliamonte MC, Catalani S, Primiterra M, Canestrari F, De Stefani S (2012). Differences in blood and semen oxidative status in fertile and infertile men, and their relationship with sperm quality. Reprod Biomed Online.

[28] Suleiman SA, Ali ME, Zaki ZMS, El-Malik EMA, Nasr MA (1996). Lipid peroxidation and human sperm motility: protective role of vitamin E. J Androl.

[29] Macanovic B, Vucetic M, Jankovic A, Stancic A, Buzadzic B, Garalejic E (2015). Correlation between sperm parameters and protein expression of antioxidative defense enzymes in seminal plasma: a pilot study. Dis Markers.

[30] Khosrowbeygi A, Zarghami N, Deldar Y (2004). Correlation between sperm quality parameters and seminal plasma antioxidants status. Iran J Reprod Med.

[31] Shiva M, Gautam AK, Verma Y, Shivgotra V, Doshi H, Kumar S (2011). Association between sperm quality, oxidative stress, and seminal antioxidant activity. Clin Biochem.

[32] Hsieh YY, Sun YL, Chang CC, Lee YS, Tsai HD, Lin CS (2002). Superoxide dismutase activities of spermatozoa and seminal plasma are not correlated with male infertility. J Clin Lab Anal.

[33] Aktan G, Doğru-Abbasoğlu S, Küçükgergin C, Kadıoğlu A, Özdemirler-Erata G, Koçak-Toker N (2013). Mystery of idiopathic male infertility: is oxidative stress an actual risk?. Fertil Steril.

[34] Atig F, Kerkeni A, Saad A, Ajina M (2017). Effects of reduced seminal enzymatic antioxidants on sperm DNA fragmentation and semen quality of Tunisian infertile men. J Assist Reprod Genet.

[35] Simon L, Proutski I, Stevenson M, Jennings D, McManus J, Lutton D (2013). Sperm DNA damage has a negative association with livebirth rates after IVF. Reprod Biomed Online.

